# Supplementary material for: The association between dietary patterns before and in early pregnancy and the risk of gestational diabetes mellitus (GDM): Data from the Malaysian SECOST cohort
Source: PLoS One. 2020 Jan 10;15(1):e0227246. doi: 10.1371/journal.pone.0227246 (PMC6953856; doi:10.1371/journal.pone.0227246)
Supplement: S1 Table — (DOCX) [file pone.0227246.s001.docx]

**S1 Table. Adjusted odd ratios and 95% confidence intervals for the associations between dietary pattern and GDM (N=452) [Sensitivity analysis]**

| **Dietary pattern** ^b^ | | **Maternal glycemia** ^a^ | | | |
| --- | --- | --- | --- | --- | --- |
|  |  | **Acceptable reporters of energy intake** | | **Under-reporters of energy intake** | |
|  |  | **Adjusted OR** | **p-value** | **Adjusted OR** | **p-value** |
| **First trimester** | DP 4 |  |  |  |  |
|  | - LA | 1.00 |  | 1.00 |  |
|  | - MA | 0.78 [0.37 – 1.63] | 0.78 | 0.82 [0.39 – 1.72] | 0.70 |
|  | - HA | 0.80 [0.36 – 1.71] | 0.80 | 0.87 [0.40 – 1.90] | 0.74 |
|  | DP 5 |  |  |  |  |
|  | - LA | 1.00 |  | 1.00 |  |
|  | - MA | 0.69 [0.34 – 1.39] | 0.26 | 0.70 [0.35 – 1.42] | 0.33 |
|  | - HA | **0.27 [0.11 – 0.67]** | **0.01^*^** | **0.28 [0.11 – 0.69]** | **0.01^*^** |
|  | DP 6 |  |  |  |  |
|  | - LA | 1.00 |  | 1.00 |  |
|  | - MA | 1.27 [0.59 – 2.72] | 0.54 | 1.27 [0.59 – 2.72] | 0.54 |
|  | - HA | 1.15 [0.54 – 2.47] | 0.72 | 1.13 [0.52 – 2.42] | 0.76 |
| **Second trimester** | DP 7 |  |  |  |  |
|  | - LA | 1.00 |  | 1.00 |  |
|  | - MA | 0.67 [0.33 – 1.38] | 0.28 | 0.62 [0.30 – 1.30] | 0.21 |
|  | - HA | 0.51 [0.24 – 1.11] | 0.08 | 0.51 [0.22 – 1.05] | 0.06 |
|  | DP 8 |  |  |  |  |
|  | - LA | 1.00 |  | 1.00 |  |
|  | - MA | 0.68 [0.32 – 1.49] | 0.34 | 0.73 [0.33 – 1.59] | 0.42 |
|  | - HA | 0.73 [0.33 – 1.57] | 0.42 | 0.86 [0.39 – 1.90] | 0.71 |
|  | DP 9 |  |  |  |  |
|  | - LA | 1.00 |  | 1.00 |  |
|  | - MA | 1.24 [0.60 – 2.55] | 0.56 | 1.39 [0.67 – 2.90] | 0.41 |
|  | - HA | 0.73 [0.33 – 1.63] | 0.44 | 0.89 [0.39 – 2.03] | 0.77 |

Note. ^a^ The reference category is non GDM.

^b^ Dietary patterns were classified in tertiles of adherence (1st tertile= low adherence (LA); 2nd tertile= moderate adherence (MA) & 3rd tertile= high adherence (HA)).

Adjusted for clinic, gestational week at OGTT performed, maternal age, ethnicity, medical history of GDM and family history of DM.

*p<0.05
