# Supplementary material for: The association between dietary patterns before and in early pregnancy and the risk of gestational diabetes mellitus (GDM): Data from the Malaysian SECOST cohort
Source: PLoS One. 2020 Jan 10;15(1):e0227246. doi: 10.1371/journal.pone.0227246 (PMC6953856; doi:10.1371/journal.pone.0227246)
Supplement: S2 Table — (DOCX) [file pone.0227246.s002.docx]

**S2 Table.** **Energy intake in first trimester of women by pre-pregnancy BMI**

| **DP 5** | **Underweight and normal weight** | **Overweight and obese** | **t** | **p-value** |
| --- | --- | --- | --- | --- |
| - LA | 1538 ± 513.52 | 1667 ± 630.66 | 2.36 | 0.02* |
| - MA | 1610 ± 604.25 | 1664 ± 582.19 | 0.53 | 0.60 |
| - HA | 1541 ± 463.87 | 1624 ± 611.37 | 2.81 | 0.03* |

*p<0.05
